# Down‐regulated LINC00115 inhibits prostate cancer cell proliferation and invasion via targeting miR‐212‐5p/FZD5/Wnt/β‐catenin axis

**DOI:** 10.1111/jcmm.17000

**Published:** 2021-10-26

**Authors:** Naixiong Peng, Zejian Zhang, Yaomin Wang, Minlong Yang, Jiqing Fan, Qinjun Wang, Ling Deng, Dong Chen, Yuefeng Cai, Qihui Li, Xisheng Wang, Wei Li

**Affiliations:** ^1^ Department of Urology Shenzhen Longhua District Central Hospital The Affiliated Central Hospital of Shenzhen Longhua District Guangdong Medical University Shenzhen China; ^2^ Wake Forest University Winston‐Salem NC USA

**Keywords:** invasion, LINC00115, miR‐212‐5p, proliferation, prostate cancer

## Abstract

Prostate cancer is the second most frequent malignancy in men worldwide, and its incidence is increasing. Therefore, it is urgently required to clarify the underlying mechanisms of prostate cancer. Although the long non‐coding RNA LINC00115 was identified as an oncogene in several cancers, the expression and function of LINC00115 in prostate cancer have not been explored. Our results showed that LINC00115 was significantly up‐regulated in prostate cancer tissues, which was significantly associated with a poor prognosis for prostate cancer patients. Functional studies showed that knockdown LINC00115 inhibited cell proliferation and invasion. In addition, LINC00115 served as a competing endogenous RNA (ceRNA) through sponging miR‐212‐5p to release Frizzled Family Receptor 5 (FZD5) expression. The expression of miR‐212‐5p was noticeably low in tumour tissues, and FZD5 expression level was down‐regulated with the knockdown of LINC00115. Knockdown LINC00115 inhibited the Wnt/β‑catenin signalling pathway by inhibiting the expression of FZD5. Rescue experiments further showed that LINC00115 inhibits prostate cancer cell proliferation and invasion via targeting miR‐212‐5p/ FZD5/ Wnt/β‐catenin axis. The present study provided clues that LINC00115 may be a promising novel therapeutic target for prostate cancer patients.

## 1 INTRODUCTION

Prostate cancer (PCa) is still one of the leading causes of cancer‐related death threatening men's health around the world.^1^ Previous studies showed that approximately 10% of newly diagnosed patients had metastatic lesions, and 5% eventually developed metastatic after surgery.^2^, ^3^ Recently years, numerous efforts have been made by researchers globally to explore the novel therapeutic strategies, and the prognosis for patients with advanced PCa remains unfavourable.^4^ The 5‐year survival rate was still not optimistic because of the lack of sensitive biomarkers in early diagnosis and effective treatments. Therefore, it is urgent to explore the molecular mechanism of PCa development and identify a novel bio‐labelling for the early diagnosis or treatment.

Increasing studies have reported that long non‐coding RNAs (lncRNAs) are longer than 200 nt without protein‐coding ability, but might play key roles in cancer cell proliferation and metastasis.^5^, ^6^ Emerging evidence has revealed that many lncRNAs are dysregulated in a variety of human cancers such as prostate cancer.^7^ Due to their expression specificity, several lncRNAs have been defined to be prostate cancer biomarkers and may be exploited for noninvasive liquid biopsy applications, such as lncRNA SChLAP1 and lncRNA PCA3.^8^, ^9^, ^10^ Lots of lncRNA may act as oncogenes or tumour suppressors in PCa. For example, lncRNA SNHG3 promoted cell proliferation and accelerated prostate cancer progression by miR‐577/SMURF1 axis.^11^ Furthermore, some lncRNA can also regulate androgen receptor (AR) signalling, which plays a central role in the progression of Castration‐Resistant Prostate Cancer (CRPC). LINC00844 was demonstrated to be a direct androgen‐regulated target that is actively transcribed in androgen receptor (AR)‐dependent prostate cancer cells.^12^ LncRNA LBCS functions as a novel AR translational regulator that suppresses castration resistance of prostate cancer by interacting with hnRNPK.^13^ Nevertheless, investigating the function and mechanism of novel lncRNAs is necessary to find functional biomarkers in PCa progression.

LINC00115 was originally identified as a long intergenic non‐protein‐coding RNA 115.^14^ However, few studies have investigated the function and mechanism of LINC00115 in cancer development and progression. For instance, LINC00115 serves as an oncogene and contributes to the progression of colorectal cancer by targeting miR‐489‐3p via the PI3K/AKT/mTOR pathway.^15^ The previous study has also reported that LINC00115 is up‐regulated and promotes breast cancer metastasis through modulating the expression of miR‐7 and KLF4.^16^ In addition, INC00115 acts as a miRNA sponge by competitively binding miR‐200s to up‐regulate ZEB1, thereby enhancing glioma stem‐like cell self‐renewal.^17^ Therefore, anti‐LINC00115 compounds or agents might serve as novel therapeutic strategies for cancer treatment. However, the functional roles of LINC00115 in prostate carcinogenesis were still unknown. In this study, the expression level of LINC00115 was measured in prostate cancer tissues and cells. We also investigated the function and mechanism of LINC00115 in PCa cells. Our findings strongly suggested that LINC00115 participates in PCa progression and is a promising therapeutic target.

## 2 MATERIALS AND METHODS

### 2.1 Clinical samples

24 paired samples (aged from 26 to 60 years old) of prostate cancer tissues and adjacent normal tissue were obtained from Shenzhen Longhua District Central Hospital (Shenzhen, China). All these experimental procedures have been approved by the Ethics Committee of Shenzhen Longhua District Central Hospital. Tissue samples were immediately preserved in liquid nitrogen at −196°C promptly. Before surgery, no patients had received chemotherapy or radiotherapy. Written informed consents were obtained from patients.

### 2.2 Cell lines and cultures

The human prostate cancer cell lines (PC‐3, DU145, LNCap and 22RV2) were purchased from the American Type Culture Collection (ATCC). Human prostatic epithelial cell (RWPE) was purchased from Shanghai Cell Bank (Chinese Academy of Sciences) and cultured in prostate epithelial cell medium (ScienCell, Carlsbad, CA, USA) containing human recombinant EGF(Gibco) and foetal bovine serum (Hyclone). All the cancer cells were cultured in DMEM (Gibco, Carlsbad, CA, USA) containing 10% foetal bovine serum.

(FBS; Gibco) at 37°C in an incubator containing 5% CO_2_.

### 2.3 Cell transfection

Firstly, PCa cells were seeded into 6‐well plates. After 24h, cell transfection was carried out according to the instruction of Lipofectamine 3000 (Invitrogen). Specific shRNAs against LINC00115 (shRNA‐1(5’‐ GAAGAAUGGUACAAAUCCAAG‐3’) and shRNA‐2 (5’‐CUUAAAGGAACCAAUGAGUCC‐3’) and their corresponding NC (Control‐shRNA) were bought from GenePharma.^18^ The pcDNA3.1 vector (pcDNA‐FZD5) and the empty vector were also acquired from GenePharma. Besides, miR‐212‐5p mimics, mimics control, miR‐212‐5p inhibitor and inhibitor control were obtained from GenePharma.^19^ Subsequently, PC‐3 and DU145 cells were severally transfected with the above plasmids. Cell transfection was conducted in triplicate. After 48 h, quantitative RT‐PCR was performed to determine the transfection efficiency.

### 2.4 Proliferation assay

After transfection, PC‐3 and DU145 cells were seeded into 96‐well plates (1000/well). After 24, 48, 72 and 94 h, 10 μl CCK‐8 solution (Beyotime) was added to each well and incubated for 1 h. Then, the absorbance (OD value at 450 nm) was measured using a microplate reader. The average of OD values from three wells in each group was calculated, and the proliferation curve was drawn.

### 2.5 Colony formation assay

Colony Formation Assay was used to further measure cell proliferation. After transfection, PC‐3 and DU145 cells were seeded into 6‐well plates (700/well) and cultured at 37°C in an incubator containing 5% CO_2_. After 14 days, cells were fixed by 4% paraformaldehyde (Beyotime) for 10 min and visualized by 0.1% crystal violet (Beyotime) for 5 min. The number of colonies (at least 50 cells) was counted with a visual inspection.

### 2.6 Cell invasion assays

Transwell inserts (8 μm‐pore sizes) coated with Matrigel (BD Biosciences) were used to measure the invasive ability of cells. After transfection, PC‐3 and DU145 cells were trypsinized and resuspended into a fresh medium containing 1% FBS. Cells (2.0 × 10^4^) were put into the upper chambers, while a medium containing 15% FBS was added into the lower chambers for chemo‐attractant. Following culture for 48 h at 37˚C, cells were fixed for 10 min by 4% paraformaldehyde (Beyotime, Beijing, China) and stained for 10 min in 0.1% crystal violet (Beyotime). Thereafter, cells on the inner layer were wiped off softly with a cotton swab. The cells were then evaluated by a light microscopy (CK40; Olympus Corporation) at the magnification of × 100. Images were captured, then the cells were counted randomly in 5 fields and the average was calculated.

### 2.7 RNA isolation and qRT‐PCR

Total RNA was isolated from prostates cancer and normal tissues and cells according to the instructions of TRIZOL Reagent (Invitrogen, USA). Then, RNA was subsequently reverse‐transcribed into cDNA by using PrimeScript 1st Strand cDNA Synthesis Kit (TaKaRa, Tokyo, Japan) as recommended by the manufacturer. qRT‐PCR was carried out with Bio‐Rad iQ5 Real‐Time PCR System SYBR Green kit (TaKaRa, Tokyo, Japan). GAPDH or U6 was used as the internal control, and relative gene expressions were determined by the 2^−^ **^△△^** ^Ct^ approach. The primers for LINC00115 and GAPDH sequences were used according to the previous report.^15^

### 2.8 Dual‐luciferase reporter assay

Firstly, PC‐3 cells were seeded into 24‐well plates (5.0 × 10^4^/Well). After 12h, pcDNA3.1‐LINC00115 Wt, pcDNA3.1‐LINC00115 Mut, miR‐212‐5p mimics or mimics control were transfected into PC‐3 cells with Lipofectamine 2000 (Invitrogen, USA). In addition, pcDNA3.1‐FZD5 Wt,pcDNA3.1‐ FZD5 Mut and miR‐212‐5p mimics and mimics control were transfected into PC‐3 cells with Lipofectamine 2000 (Invitrogen).After 24 h, cells were harvested for luciferase detection using the Dual‐Luciferase Reporter assay system (Promega).

### 2.9 RNA immunoprecipitation assay

The Magna RIP™ RNA‐Binding Protein Immunoprecipitation kit (Millipore Corp, Atlanta, GA, USA) was used for RNA immunoprecipitation (RIP) experiments. Firstly, PC‐3 cells were collected (>10^7^ cells) by trypsinization and resuspended in PBS. Then, cells were lysed in freshly prepared RIP buffer. Cell lysate (100 μl) was incubated with RIP buffer containing magnetic beads conjugated with Ago2 antibody (Abcam) or negative control IgG. The samples were incubated with proteinase K with shaking to digest proteins, and then, the precipitation of RNA was obtained. Co‐precipitated RNA was measured by RT‐PCR.

### 2.10 Western Blot

Total protein was extracted as described previously.^20^, ^21^ Briefly, radioimmunoprecipitation assay (RIPA) lysis buffer (Beyotime, Shanghai, China) was used to extract the total proteins. Subsequently, protein concentration was determined with the BCA Protein Assay Kit (Beyotime). Then, equal quantities of proteins were uploaded and separated on 12% sodium dodecyl sulphate polyacrylamide gel electrophoresis (SDS‐PAGE) gel and transferred into polyvinylidene fluoride membranes (PVDF) (Millipore Corp). Then, the membranes were incubated with 5% non‐fat dry milk powder at room temperature for 2 h and then incubated with primary antibodies as follows: anti‐N‐cadherin (Abcam,1:1000), anti‐vimentin (Abcam, 1:1000), anti‐E‐cadherin (CST,1:800), anti‐FZD5(Abclonal,1:800), anti‐β‐catenin(Abcam, 1:800) and anti‐cyclin D1(Abcam, 1:1000) or overnight at 4°C, followed by HRP‐conjugated secondary antibodies at room temperature for 1 h. The housekeeper gene GAPDH was employed as an internal control. The signals of bands were detected by ECL reagents.

### 2.11 Statistical analysis

All data were expressed as mean ± standard deviation (S.D). SPSS 19.0 (SPSS) was used to conduct all the statistical analyses. Student's *t* test was used to evaluate the differences between variables, and the *p* value less than 0.05 was regarded as statistically significant.

## 3 RESULTS

### 3.1 LINC00115 was up‐regulated in PCa and correlates with poor prognosis

**FIGURE 1:**
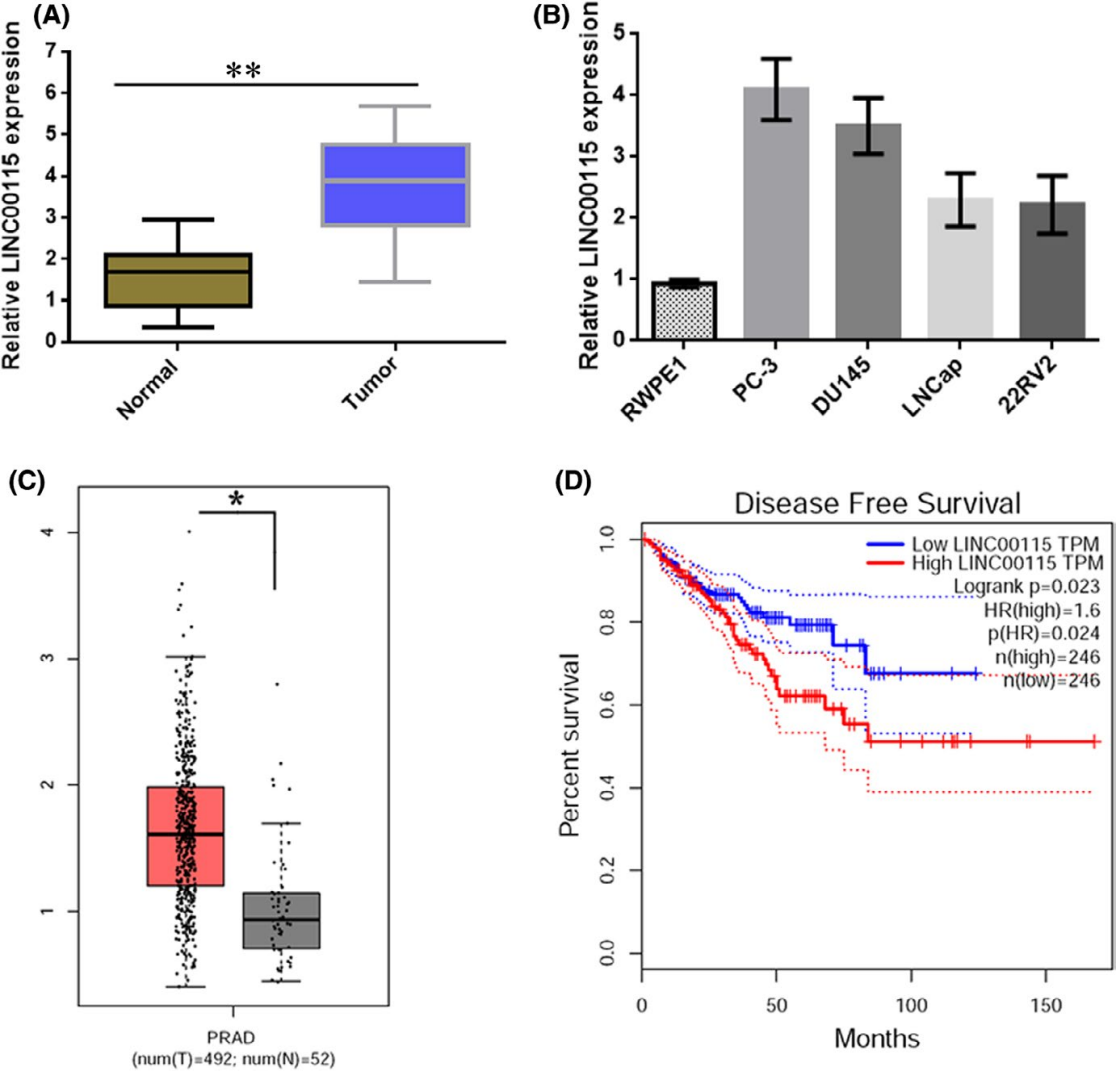
LINC00115 is highly expressed in prostate cancer tissues. (A) The expressions of LINC00115 in prostate cancer tissues and normal tissues were analysed by qRT‐PCR. (B) Relative LINC00115 expression in prostate cancer cell line (PC‐3, DU145, LNCap and 22RV2) and Human prostatic epithelial cell (RWPE) was measured by RT‐PCR. (C) The expressions of LINC00115 in prostate tumour and normal tissues were analysed based on TCGA database, T: tumour tissues, N: normal tissues. (D) Kaplan‐Meier survival curves showed that LINC00115 expression level was negatively correlated with prognosis prediction of prostate cancer analysed by TCGA database. *p < 0.05, **p < 0.01

To explore the role of LINC00115 in prostate cancer, we firstly compared LINC00115 expression between normal tissues and PCa tissues by qRT‐PCR. The results showed that the expression of LINC00115 was significantly up‐regulated in PCa tissues (Figure 1A). In addition, it was found that LINC00115 was significantly up‐regulated in prostate cancer cell lines, LNCaP, PC‐3, DU145 and 22RV2 (Figure 1B). Overall, LINC00115 was up‐regulated in prostate cancer tissues and cell lines. PC‐3and DU145 cells were selected for the later investigations because of high LINC00115 expression. Moreover, gene expression data from TCGA database demonstrated that LINC00115 expression was substantially higher in PCa tissues than that in normal tissues (Figure 1C). Kaplan‐Meier survival analysis showed that the disease‐free survival of LINC00115 high expression group was shorter than that of LINC00115 low expression group (Figure 1D). Taken together, these findings indicate that up‐regulation of LINC00115 may play an important role in PCa tumorigenesis.

### 3.2 Knockdown LINC00115 inhibits prostate cancer growth in vitro

**FIGURE 2:**
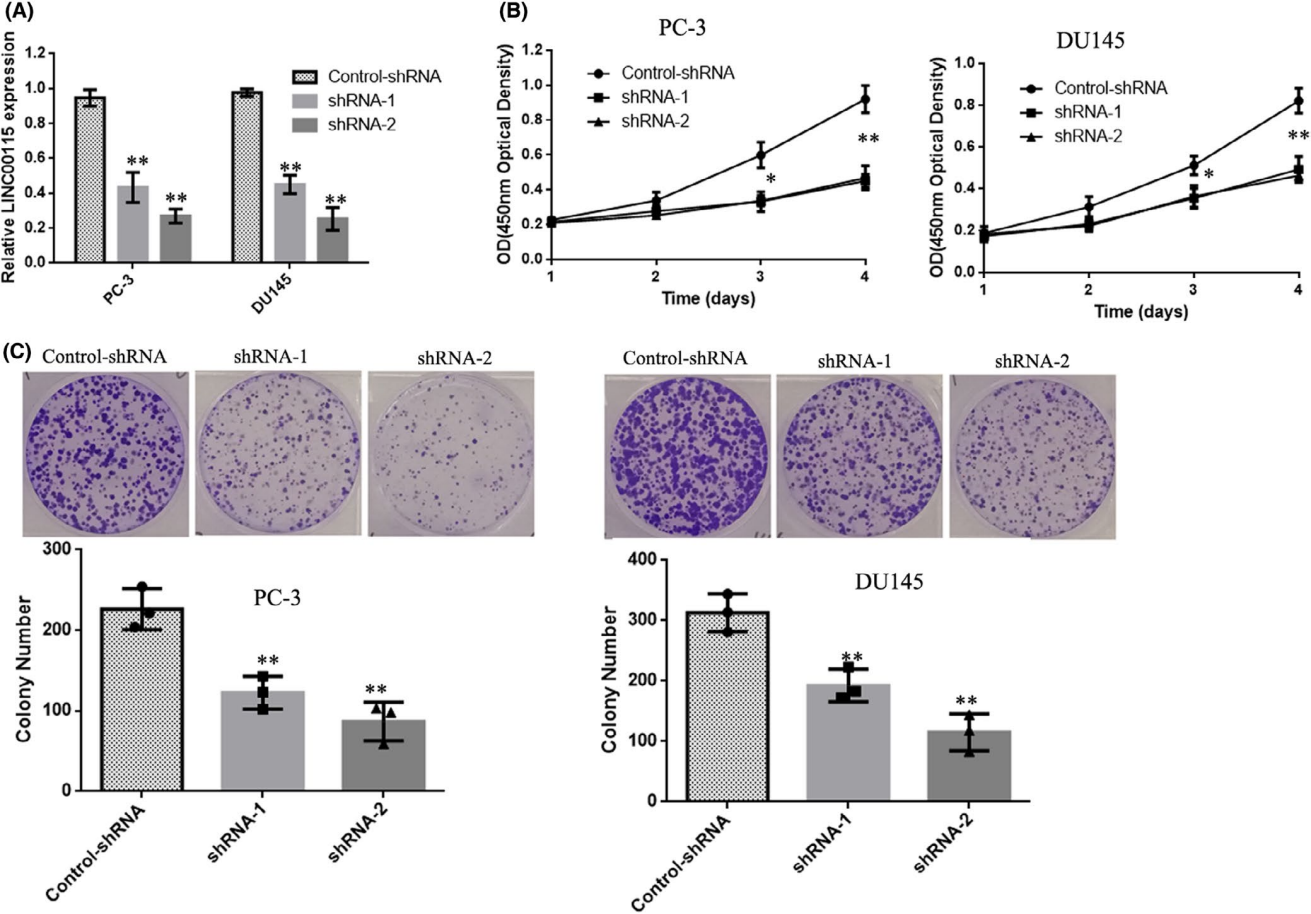
Knockdown LINC00115 inhibits the proliferation of prostate cancer cells in vitro. (A) PC‐3 and DU145 cells transfected with shRNA‐1, shRNA‐2 or Control‐shRNA. After 48 h, the transfection efficiency confirmed by qRT‐PCR. (B) The cell viability was measured using CCK‐8 assay. (C) Colony formation assay of PC‐3 and DU145 cells transfected with shRNA‐1, shRNA‐2 or Control‐shRNA. After incubation for 14 days, colonies were stained and photographed. Data are presented as the mean ± SD of three independent experiments. **p < 0.01, *p < 0.05

To further investigate the role of LINC00115 in PCa progression, two high‐efficiency targeted shRNAs were stably transfected into two PCa cell lines (PC‐3and DU145) that highly expressed LINC00115. The results showed that compared with a negative control group (Control‐shRNA), the selected shRNAs (shRNA‐1 and shRNA‐2) could significantly down‐regulate LINC00115 expression in PC‐3 and DU145 (Figure 2A). Cell growth assays showed that knockdown LINC00115 can significantly inhibit proliferation capabilities (Figure 2B). Furthermore, the colony formation assay demonstrated that knocking down LINC00115 significantly reduced the number of colonies when compared with control cells (Figure 2C). These data indicated that knockdown LINC00115 inhibited prostate cancer cells proliferation.

### 3.3 Knockdown LINC00115 inhibits prostate cancer cell invasion in vitro

**FIGURE 3:**
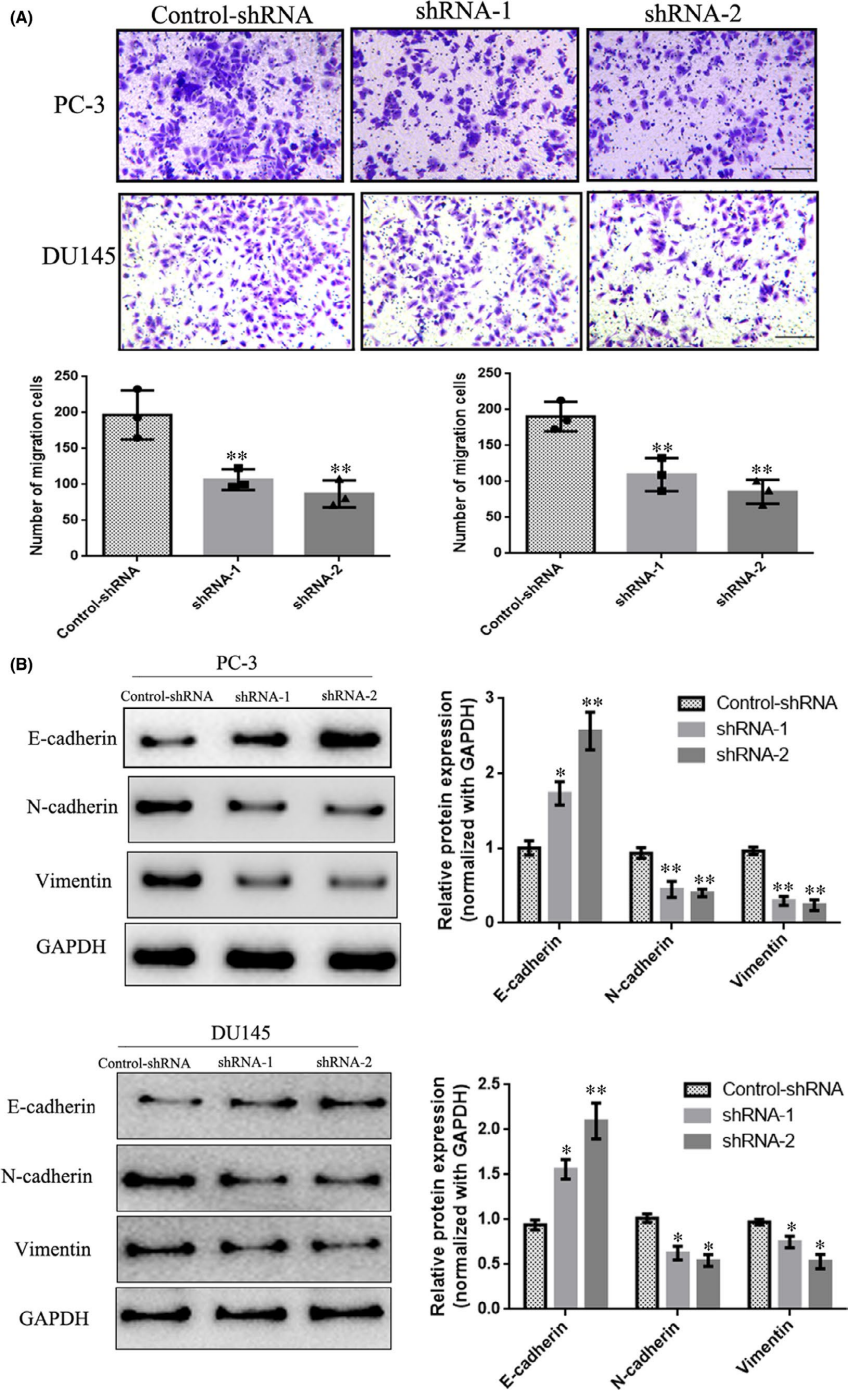
Knockdown LINC00115 inhibits prostate cancer invasion. (A) Invasion assays were measured using Transwell champers. (B) Knockdown LINC00115 inhibited EMT in prostate cancer cells. Data represent mean ± S.D of three independent experiments. Scale bar = 100 μm, *p < 0.05, **p < 0.01

Transwell invasion assay was used to measure the effect of LINC00115 on the invasion of PCa cells. The results showed that knockdown LINC00115 significantly decreased the invasion ability of PC‐3 and DU145 cells (Figure 3A). Additionally, Western blot showed that knockdown LINC00115 could significantly increase E‐cadherin expression, whereas decrease the mesenchymal markers (N‐cadherin and vimentin) expression (Figure 3B). These data indicate that knockdown LINC00115 can inhibit prostate cancer cell invasion by inhibiting EMT.

### 3.4 LINC00115 directly interacts with miR‐212‐5p in prostate cancer

**FIGURE 4:**
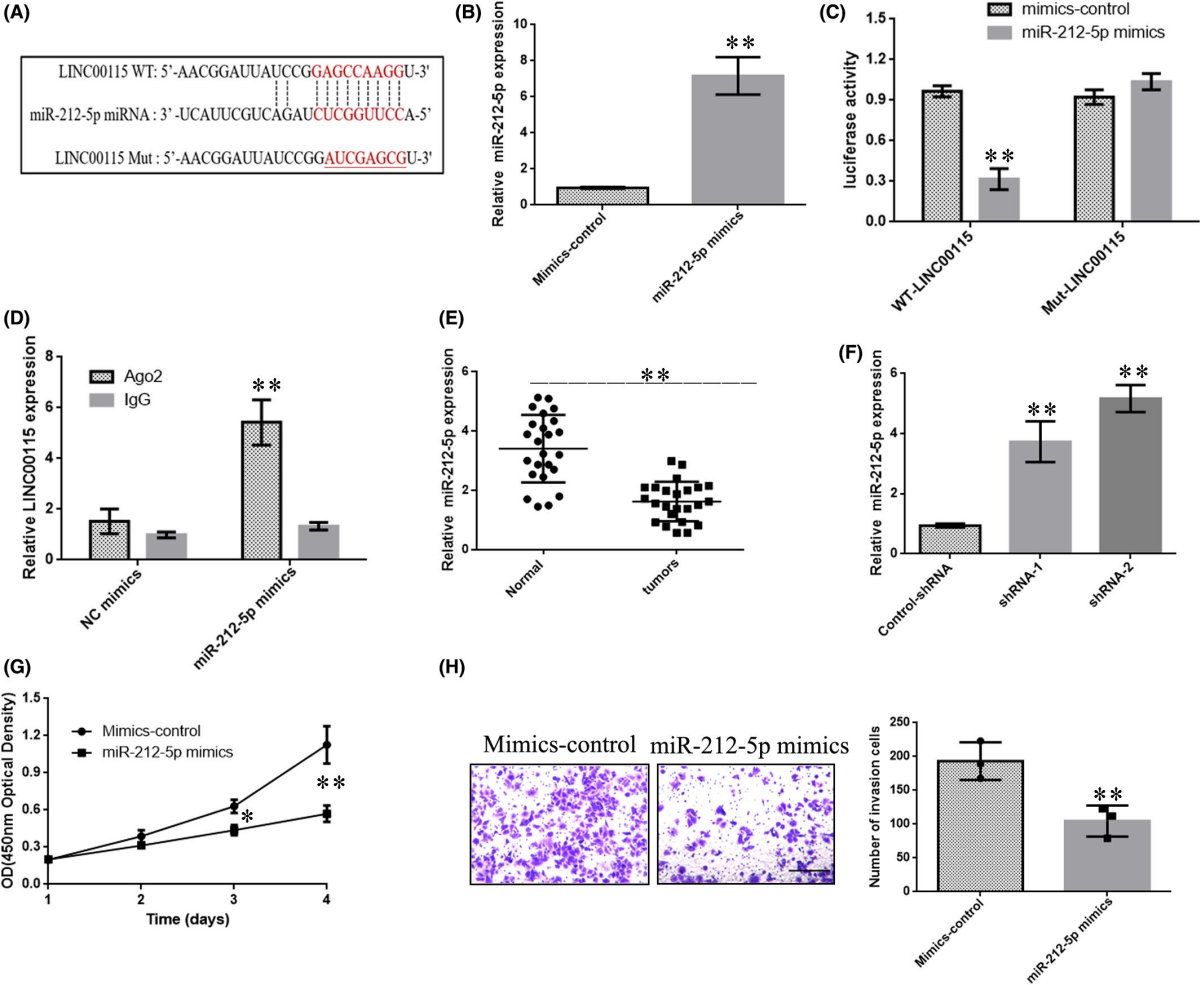
LINC00115 directly interacts with miR‐212‐5p. (A) The potential binding region with miR‐212‐5p in LINC00115 and the mutant form of LINC00115 were shown. (B) miR‐212‐5p mimics and control mimics were transfected into PC‐9 cells. (C) The luciferase intensity of PC‐9 cells transfected with LINC00115 wild type and miR‐212‐5p was significantly reduced. (D) RIP was performed in PC‐9 cells by using anti‐Ago2. LINC00115 expression was detected using qRT‐PCR. (E) miR‐212‐5p expression in prostate cancer tissues and adjacent normal samples was tested by qRT‐PCR. (F) Knockdown LINC00115 up‐regulated the expression of miR‐212‐5p in PC‐3 cells. (G) CCK‐8 assay was used to measure the effect of ectopic expression of miR‐212‐5p. (H) Transwell assay was used to measure the effect of ectopic expression of miR‐212‐5p. Scale bar = 100 μm. Data are presented as means ± SD of three independent experiments. *p < 0.05, **p < 0.01

To further measure the downstream mechanism of LINC00115, we searched the Starbase database and found that LINC00115 had a potential binding site with miR‐212‐5p (Figure 4A). In order to verify whether LINC00115 can directly adsorb miR‐212‐5p, a dual‐luciferase reporter assay was performed. The results showed that the luciferase activity of wild‐type LINC00115 was markedly reduced by miR‐212‐5p mimics, while the luciferase activity of the mutated LINC0011 was not changed after co‐transfecting with miR‐212‐5p mimics (Figure 4C). RIP assay was performed in PC‐3 cells by using the antibody against Ago2. Compared with mimics control, the endogenous LINC00115 was specifically enriched in the cells of miR‐212‐5p mimics, indicating that LINC00115 directly targeted miR‐212‐5p (Figure 4D). In addition, we found that miR‐212‐5p was significantly down‐regulated in PCa samples (Figure 4E). Moreover, the expression of miR‐212‐5p was significantly up‐regulated after knockdown LINC00115 (Figure 4F). Collectively, these data indicated that miR‐212‐5p might be a direct target of LINC00115 in prostate cancer. In addition, to investigate the role of miR‐212‐5p in PCa, the transfection of miR‐212‐5p mimics could inhibit the proliferation of PC‐3 cells (Figure 4G). Transwell assay showed that the transfection of miR‐212‐5p mimics significantly reduced the invasion of PC‐3 cells (Figure 4H). These results consistently indicated that down‐regulation of LINC00115 might suppress prostate cancer cell growth by targeting miR‐212‐5p.

### 3.5 LINC00115 functions as a ceRNA through sponging miR‐212‐5p to up‐regulate FZD5

**FIGURE 5:**
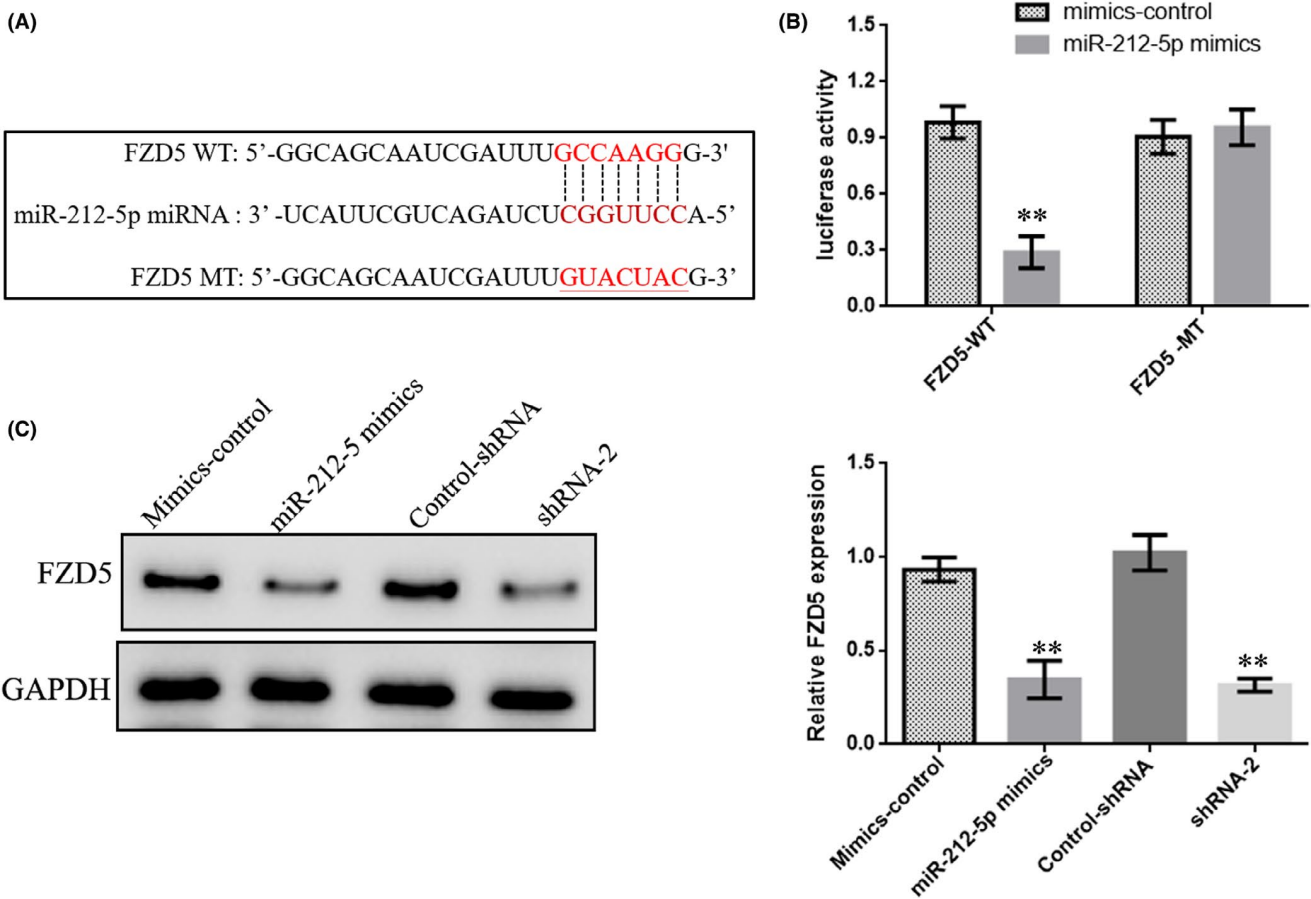
LINC00115 functions as a ceRNA through sponging miR‐212‐5p to regulate FZD5. (A) The binding sites between miR‐212‐5p and FZD5 obtained from starBase database. (B) The luciferase reporter was implemented to determine the binding correlation between miR‐212‐5p and FZD5 in PC‐3 cells. (C) Western blot assay was performed to detect the expression of FZD5 in PC‐3 cells after transfected with miR‐212‐5p mimics or shRNA‐2(LINC00115). Data are presented as means ± SD of three independent experiments. *p < 0.05, **p < 0.01

Previous studies have demonstrated that miRNA exerts their multiple biological functions mainly by suppressing mRNA translation or degrading mRNA. By querying TargetScan database, the results showed that 3’UTR of FZD5 contained a potential binding site with miR‐212‐5p (Figure 5A). Subsequently, the luciferase report assay confirmed that miR‐212‐5p could bind to the 3’UTR of FZD5 (Figure 5B). To further measure the relationship between the expression of FZD5 and miR‐212‐5p expression in PCa, Western blot showed that the expression of FZD5 was decreased significantly after the transfection of miR‐212‐5p mimics, while Knockdown LINC00115 also decreased FZD5expression.

**FIGURE 6:**
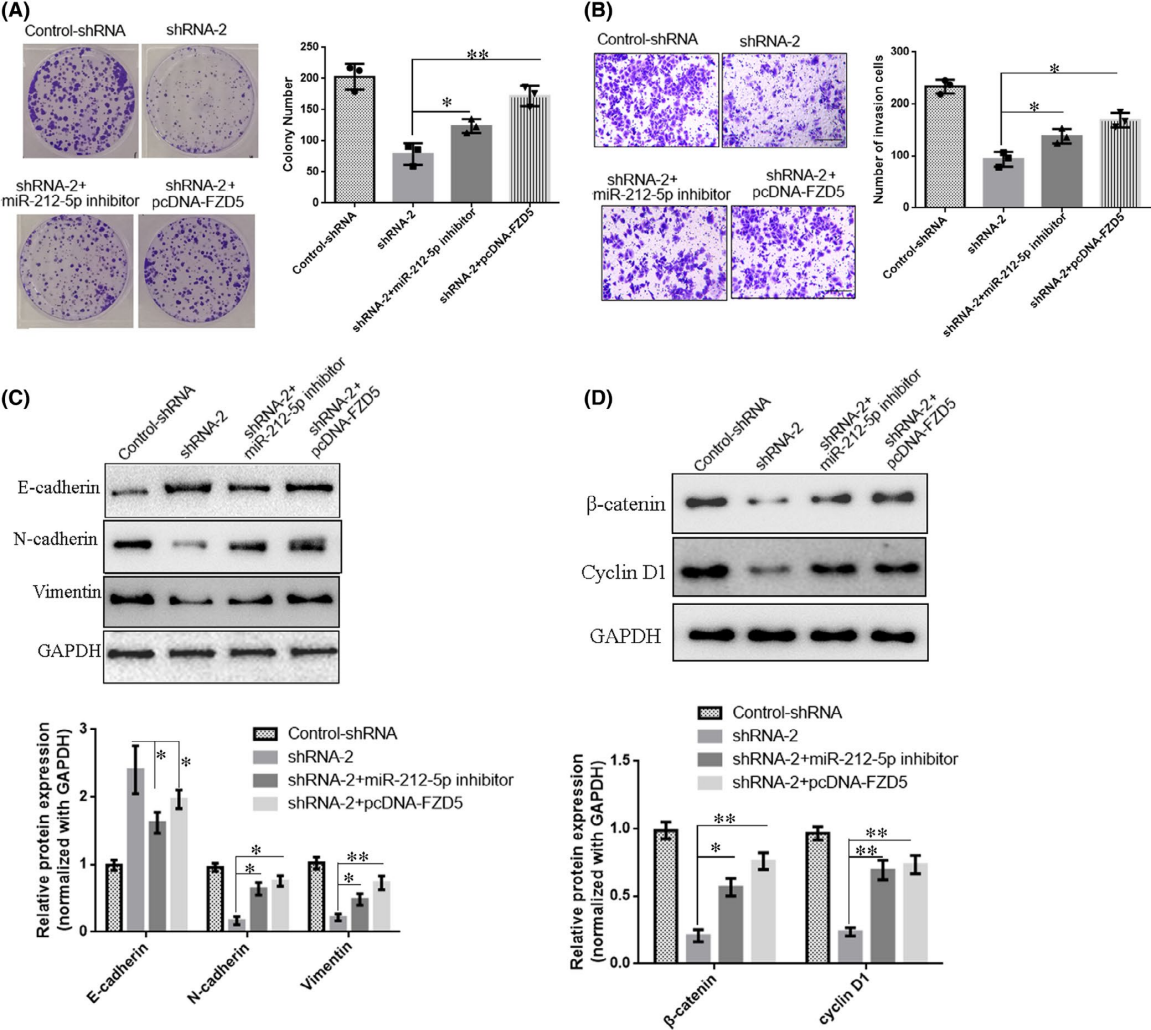
LINC00115 inhibits prostate cancer cell proliferation and invasion via targeting miR‐212‐5p/ FZD5 axis. (A, B) Colony formation assay and Transwell assay were utilized to assess cell proliferation and invasion, respectively. Scale bar = 100 μm. (C) EMT process in prostate cells was evaluated by Western blot. (D) Western blot was used to further determine the effects of FDZ5 on the expression of relative factors (β‐catenin and cyclin D1) in Wnt/β‐catenin signal pathways. Data are presented as means ± SD of three independent experiments. *p < 0.05, **p < 0.01

In order to verify the role of LINC00115/miR‐212‐5p/ FZD5 axis in PCa progression, LINC00115 shRNA(shRNA‐2), miR‐212‐5p inhibitor and pcDNA‐FZD5 were co‐transfected into PC‐3 cells. The functional assays showed that knockdown of LINC00115 inhibited PC‐3 cells growth and invasion, while co‐transfection of miR‐212‐5p inhibitors restored these results. In addition, compared with LINC00115 shRNA group, overexpressing FZD5 (pcDNA‐FZD5) abolished the inhibition of knockdown LINC00115 on PC‐3 cells growth and invasion (Figure 6A,B). Simultaneously, Western blot revealed that EMT progression inhibited by silencing LINC00115 was partially restored by the up‐regulation of FZD5 or miR‐212‐5p inhibitors (Figure 6C).

Wnt/β‐catenin is a key molecular signalling regulator in a variety of human cancers and is usually triggered through the secreted Wnt ligands binding to Frizzled (FZD) receptor proteins, including FZD5. Therefore, a Western blot was used to further determine the effects of FDZ5 on the expression of relative factors (β‐catenin and cyclin D1) in Wnt/β‐catenin signal pathways. The results showed compared with control‐shRNA, knockdown of LINC00115 significantly inhibited the expression of β‐catenin and cyclin D1, while the inhibitory effects of knockdown LINC00115 on β‐catenin and cyclinD1 expression were reversed after co‐transfecting with miR‐212‐5p inhibitors or pcDNA‐FZD5 (Figure 6D). Collectively, these data suggested that down‐regulated LINC00115 inhibits prostate cancer cell proliferation and invasion via targeting miR‐212‐5p/ FZD5/ Wnt/β‐catenin axis.

## 4 DISCUSSION

Recently, an increasing number of researches has revealed the crucial role of lncRNAs in various biological functions and disease processes including cancer.^6^, ^7^ They act as a multifunctional regulator to promote or inhibit tumour growth by various mechanisms such as transcriptional, posttranscriptional and epigenetic levels.^22^, ^23^, ^24^ In this present study, we identified that LINC00115 was up‐regulated in TCGA PCa samples and collected PCa samples. High expression of LINC00115 is correlated with the overall survival of patients with PCa. We further analysed the role of LINC00115 in PCa cell proliferation, invasion and explored the molecular mechanisms.

In the past decades, studies on molecular mechanisms of PCa have mainly focussed on the genome mutations, or the oncogenes or tumour suppressor genes that coded proteins as well as epigenetic changes. To date, more and more reports proved that lncRNA played a crucial role in human tumorigeneses, such as proliferation and metastasis. For example, LncRNA DRAIC inhibited proliferation and metastasis of prostate cancer by interacting with IKK to inhibit NF‐κB activation.^25^ In the current study, we found that LINC00115 is up‐regulated PCa tissues and cell lines. Furthermore, overexpression of LINC00115 was significantly associated with a poor prognosis for PCa patients. These results indicated that LINC00115 might be a potential prognostic biomarker in PCa. Functionally, knockdown of LINC00115 inhibited PCa cell proliferation and clone formation. Furthermore, knockdown of LINC00115 significantly inhibited cell invasion by increasing the expression of E‐cadherin and reducing the expression of N‐cadherin and vimentin. Our results were consistent with previous studies that knockdown of LINC00115 inhibits cancer cell (breast cancer, ovarian cancer and colorectal cancer) proliferation and invasion.^15^, ^16^, ^18^

It was well known that lncRNAs serve as competing endogenous RNAs (ceRNAs) through competing with mRNAs to bind with miRNAs. For instance, LINC00115 can function as a ceRNA by acting as miR‐30a sponge to indirectly regulate SOX9 expression and promote stemness of ovarian cancer.^18^ LINC00115 forms an miR‐7/KLF4 pathway to promote breast cancer metastasis.^16^ Based on bioinformatics analysis and mechanism assays, we found that LINC00115 could bind with miR‐212‐5p and knockdown LINC00115 increased the miR‐212‐5p expression. This result was not consistent with the previous report,^15^, ^16^, ^18^ and LINC00115 has a different miRNA binding target in different cancer types. This might be the result of tumour heterogeneity or different genetic backgrounds. It is well known that cancer cell heterogeneity is a main challenge in cancer medicine and exerts distinct effects (oncogenes or tumour suppressor genes) on tumour cells and tumour microenvironment.^26^, ^27^

miR‐212‐5p was reported to inhibit the malignant behaviour of clear cell renal cell carcinoma cells by targeting TBX15.^28^ miR‐212‐5p also exerts tumour promoter function by regulating the Id3/PI3K/Akt axis in lung adenocarcinoma cells.^29^ However, miR‐212‐5p suppresses the epithelial‐mesenchymal transition in triple‐negative breast cancer by targeting Prrx2.^30^ These results showed that miR‐212‐5p may function as carcinogens or tumour suppressors in different tumours. In this study, we found that overexpression miR‐212‐5p significantly inhibited cell proliferation and invasion. These results were further supported that the same miRNA may play opposing roles in different processes.

miRNA is a small non‐coding RNA molecule that functions as translational repression by degrading the protein‐coding mRNA. So we further identified that frizzled class receptor 5 (FZD5) as the downstream target of miR‐212‐5p. Previous studies showed that knockdown FZD5 gene can inhibit migration, invasion and bone metastasis of prostate cancer cells.^31^ In addition, our results showed that overexpression miR‐212‐5p or the knockdown LINC00115 can significantly inhibit FZD5 expression. The further rescue assays showed that FZD5 restores the effects of knockdown LINC00115 on cell proliferation, invasion and EMT‐related protein expression.

Wnt/β‐catenin is a key molecular signalling regulator in a variety of human cancers and is usually triggered through the secreted Wnt ligands binding to Frizzled (FZD) receptor proteins, including FZD5.^32^, ^33^ Knockdown FZD5 can promote cellular senescence by inhibiting the noncanonical Wnt pathway.^34^ Circ_0067934 directly suppressed miR‐1324, which targeted the 3'‐UTR of FZD5 mRNA and subsequently down‐regulated the Wnt/β‐catenin signalling pathway in hepatocellular carcinoma.^35^ Furthermore, the Wnt/β‐catenin signalling pathway plays a crucial role in regulating EMT.^36^, ^37^ Our results were consistent with these reported that knockdown of LINC00115 significantly inhibited the expression of β‐catenin and cyclin D1, while the inhibitory effects of knockdown LINC00115 on β‐catenin and cyclinD1 expression were reversed after co‐transfecting with miR‐212‐5p inhibitors or pcDNA‐FZD5. Collectively, these data suggested that down‐regulated LINC00115 inhibits prostate cancer cell proliferation and invasion via targeting miR‐212‐5p/ FZD5/ Wnt/β‐catenin axis.

## 5 CONCLUSION

In conclusion, our study demonstrated the LINC00115 is aberrantly over‐expressed in PCa tissues and cells lines. Knocking down LINC00115 can significantly reduce PCa cell proliferation and invasion. We also innovatively uncovered that LINC00115 endogenously sponged miR‐212‐5p to elevate FZD5 expression, thus facilitating the malignant phenotype of prostate cancer, which might provide new insight into identifying the biomarkers for prostate cancer.

## CONFLICT OF INTEREST

The authors confirm that there are no conflicts of interest.

## AUTHOR CONTRIBUTIONS

**Naixiong Peng:** Data curation (equal); Investigation (equal); Methodology (equal); Validation (equal); Writing‐original draft (equal); Writing‐review & editing (equal). **zejian Zhang:** Formal analysis (equal); Software (equal); Supervision (equal). **Yaomin Wang:** Software (equal); Validation (equal). **Minlong Yang:** Investigation (equal); Methodology (equal); Writing‐original draft (equal). **Jiqing Fan:** Project administration (equal); Resources (equal); Validation (equal). **Qinjun Wang:** Formal analysis (equal); Supervision (equal); Validation (equal). **Ling Deng:** Investigation (equal); Writing‐original draft (equal); Writing‐review & editing (equal). **Dong Chen:** Project administration (equal); Software (equal); Visualization (equal); Writing‐original draft (equal). **YueFeng Cai:** Data curation (equal); Software (equal); Supervision (equal). **Qihui Li:** Formal analysis (equal); Visualization (equal); Writing‐review & editing (equal). **Xisheng Wang:** Funding acquisition (equal); Software (equal); Supervision (equal); Writing‐review & editing (equal). **Wei Li:** Formal analysis (equal); Funding acquisition (equal); Project administration (equal); Writing‐review & editing (equal).

## ETHICS APPROVAL AND CONSENT TO PARTICIPATE

The study was approved by the Ethics Committee of the Department of Shenzhen Longhua District Central Hospital, and written informed consent was obtained from each participant.

## Data Availability

The data that support the findings of this study are available from the corresponding author upon reasonable request.
